# Short-term nutrition and growth indicators in 6-month- to 6-year-old children are improved following implementation of a multidisciplinary community-based programme in a chronic conflict setting

**DOI:** 10.1017/S1368980019002969

**Published:** 2019-11-07

**Authors:** Hambardzum Simonyan, Aelita Sargsyan, Arin A Balalian, Karapet Davtyan, Himanshu A Gupte

**Affiliations:** 1 Fund for Armenian Relief of America, Yerevan, Armenia; 2 Columbia University, Mailman School of Public Health, 722 West 168th Street, New York, NY 10032, USA; 3 TB Research and Prevention Center NGO, Yerevan, Armenia; 4 Narotam Sekhsaria Foundation, Mumbai, India

**Keywords:** Stunting, Child growth, Armenia, Evaluation, Multidisciplinary intervention

## Abstract

**Objective:**

We investigated short- and long-term indicators of malnutrition and diet before and after the community-based ‘Breaking the Cycle of Poverty’ multidisciplinary intervention.

**Design:**

A historically and geographically controlled study using data collected in 2013 and 2016. We compared the prevalence of short-term indicators (anaemia, breast-feeding duration and minimum dietary diversity) and long-term indicators (stunting and wasting) in exposed communities at two time points. We then compared these factors in geographic areas exposed or not exposed to intervention. We conducted logistic regression analyses on the 2016 sample to measure associations between living in intervention communities and child growth indicators.

**Setting:**

Berd region, a chronic conflict zone near the north-eastern border of Armenia and Azerbaijan.

**Participants:**

Children aged 6 months to 6 years.

**Results:**

Analyses included data from 2013 comprising 382 children, and data from 2016 comprising 348 children living in communities where the programme was implemented, and 635 children from unexposed communities. Anaemia prevalence in exposed communities was significantly lower in 2016 *v*. 2013 (10·9 *v*. 19·1 %, *P* < 0·01). Minimum dietary diversity (79·0 *v*. 68·1 %, *P* < 0·001) and breast-feeding duration (13·0 *v*. 11·5 months, *P* < 0·002) were significantly improved in exposed communities. Prevalences of stunting (11·5 *v*. 10·2 %, *P* = 0·57) and wasting (4·8 *v*. 2·0 %, *P* = 0·07) were not significantly different. Odds of anaemia were significantly lower (OR = 0·24, 95 % CI 0·16, 0·36) in intervention communities.

**Conclusions:**

Exposure to a community-based multidisciplinary intervention reduced the rate of anaemia and improved dietary indicators.

The Sustainable Development Goals have global targets of reducing the number of children under 5 years of age who are stunted by 40 % and of reducing and maintaining childhood wasting to less than 5 % by 2025. For these goals and targets to be met by all nations and for all segments of society, it is imperative to first reach those who are the furthest behind, thus ‘leaving no one behind’^(^ ^1^ ^)^.

Malnutrition continues to be a matter of global concern, as rates of stunting are declining very slowly and wasting still affects many children^(^ ^2^ ^,^ ^3^ ^)^. The global prevalence of anaemia, another indicator of malnutrition and poor health, has decreased by only 12 % between 1995 and 2011^(^ ^4^ ^)^. Child malnutrition remains a persistent challenge, especially in conflict zones, where children are more prone to adverse health conditions^(^ ^5^ ^)^. Studies conducted in Africa, the Middle East and Mexico show that living in a conflict zone can severely affect child health, resulting in acute and chronic malnutrition and leading to disease and death through various mechanisms including insufficient food security, problems with sanitation, poor health care, diarrhoeal diseases, and internal displacement of people which deprives them of opportunities for farming^(^ ^6^ ^–^ ^9^ ^)^. Food insecurity can manifest as low dietary diversity^(^ ^10^ ^,^ ^11^ ^)^, which is associated with stunting^(^ ^12^ ^–^ ^14^ ^)^. Several other factors, such as breast-feeding duration, are assumed to be determinants of malnutrition^(^ ^15^ ^,^ ^16^ ^)^. A study conducted in Ukraine has found that mothers have ceased breast-feeding their children as a result of stress from the ongoing conflict^(^ ^17^ ^)^.

For more than 20 years Armenia has been in a situation of ‘chronic frozen conflict’^(^ ^18^ ^)^ with neighbouring Azerbaijan, situated to the east of Armenia. The communities of Tavush Province have been affected by this conflict as a result of their proximity to the conflict region and the conflict has negatively impacted all aspects of community development in the area^(^ ^19^ ^)^. The Berd region of Tavush is one of the areas closest to the line of contact and thus one of the regions that suffers most from the intermittent skirmishes^(^ ^19^ ^)^.

In 2013, the Fund for Armenian Relief (FAR), a humanitarian non-governmental organization, initiated a programme called ‘Breaking the Cycle of Poverty’ (BCPP). BCPP is a multidisciplinary community development programme implemented in the Berd region (Fig. 1). The programme mainly targets children with the goal of promoting their overall growth and development. FAR planned the multidisciplinary intervention based on the findings of a baseline survey conducted in 2013 in the target communities^(^ ^20^ ^)^. BCPP includes food supplementation and capacity building at the community level, as well as other components aimed at improving child growth and development (Box 1).

**Fig. 1:**
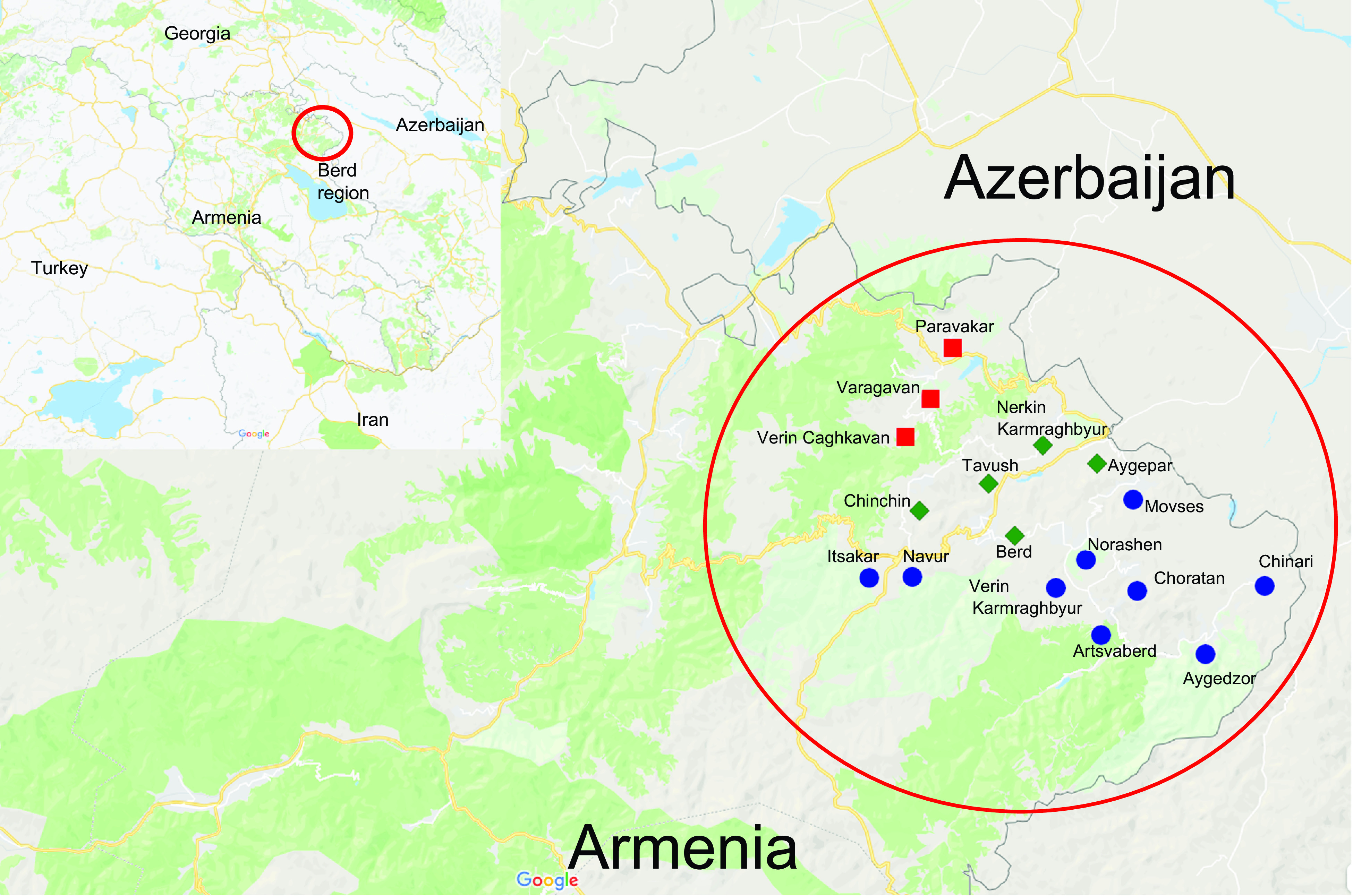
Map of Armenia, with the location of the Berd region (study region) circled.
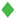
represents communities that were included in the baseline assessment in 2013 and the 2016 survey;
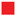
represents communities that were included in the Fund for Armenian Relief (FAR) programme in the 2013 baseline study and later excluded from the programme;
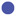
represents communities that were included in the FAR programme in 2016 and were included in the 2016 survey^(^ ^58^ ^)^

Box 1 Description of the community-based multidisciplinary programme implemented in Tavush Province, Armenia

In 2013, the Fund for Armenian Relief (FAR) implemented a comprehensive community-based programme in Tavush Province, Armenia. The primary focus of this multidisciplinary programme was the health of pre-school children. The programme included four main components:

1. improving the health of pre-school children and women of reproductive age;
2. social and psychological support for vulnerable families;
3. financial and technical assistance to local small businesses; and
4. providing scholarships to youth and developing local educational facilities.

One principal goal of the programme’s multidimensional health-care component was improving the health of 6-month to 6-year-old children in the targeted areas. This component included:

1. Continuing professional development for health-care providers, including on- and off-site opportunities for continuing medical education and training.
2. Establishment of community-based parental rooms where continuous community training on child health and nutrition was offered.
3. Improvement and strengthening of community health-care facilities, including renovations and provision of new medical equipment and supplies.
4. Improvement of pre-school facilities, including renovation and provision of kitchen supplies and appliances, as well as training for the cooks.
5. Provision of balanced food for pre-schools, including provision of fresh vegetables, fruits, dairy and meat to kitchens at kindergartens. Pre-school menus were developed in cooperation with specialists from the Armenian Ministry of Health and included necessary energy, protein, fat and carbohydrate contents. The children ate three meals per day in the pre-school setting.
6. Health education programmes targeting mothers of the children offered through pre-school facilities in the intervention communities. Topics included but were not limited to prenatal feeding, breast-feeding, complementary feeding, sanitary practices and appropriate hygiene.
7. Treatment of children diagnosed with anaemia and parasitic diseases by provision of iron supplements and antiparasitic medications.

Although similar programmes have been implemented in other countries, few are as large in scope as the FAR programme. Most other programmes have only provided food at the community level, only provided counselling or nutrition training, or targeted a different age group (e.g. teenagers)^(^ ^21^ ^,^ ^22^ ^)^. The programme implemented by FAR is unique in its multidisciplinary nature, inclusion of a school feeding programme, as well as its provision of nutritional and community health education in pre-school facilities and its collaboration with the government of Armenia. No other programme of this type has been implemented elsewhere in Armenia. Similar interventions in other countries have not included health education in pre-school facilities^(^ ^23^ ^,^ ^24^ ^)^.

In the present study, we assessed the effect of this multidisciplinary programme on growth indicators among children aged 6 months to 6 years living in communities within a conflict zone of Armenia. The specific objectives were to: (i) compare the prevalence of short-term and long-term indicators of nutrition and growth, including anaemia, mean breast-feeding duration and dietary diversity, as well as wasting and stunting, in communities exposed to the FAR programme from 2013 to 2016 with that in communities not exposed to the programme; (ii) measure changes in the prevalence of short-term and long-term indicators of growth and nutrition among children aged 6 months to 6 years living in communities that received the intervention between 2013 and 2016; and (iii) determine whether living in the intervention communities was associated with not being stunted/anaemic relative to living in the non-intervention communities.

## Methods

### Study design

This historically controlled study included data collected from a 2013 baseline survey as well as follow-up data collected in 2016^(^ ^20^ ^)^. Participants were children aged 6 months to 6 years. Measurements included Hb, height and weight, as well as a self-administered survey given to children’s mothers.

### Study setting

The 2013 baseline study was conducted in the Berd region, namely the city of Berd and seven surrounding rural communities: Nerkin Karmiraghbyur, Aygepar, Tavush, Chinchin, Verin Caghkavan, Varagavan and Paravakar villages^(^ ^20^ ^)^. In 2016, nine new rural communities were included in the FAR programme; however, three communities dropped out of the programme due to a lack of interest from the local administration and so were not included in the current assessment. The final study sample includes children living in the fourteen communities remaining in the programme in 2016: Berd city, Nerkin Karmiraghbyur, Aygepar, Tavush, Chinchin, Movses, Verin Karmiraghbyur, Choratan, Norashen, Navur, Itsakar, Artsvaberd, Chinari and Aygedzor. To compare child growth and feeding indicators before and after the intervention, we used baseline data collected in 2013 from Berd city and four rural areas (Nerkin Karmraghbyur, Aygepar, Chinchin and Tavush) as a historical control group (Fig. 1).

### Sampling frame, strategy and sample size

**Fig. 2:**
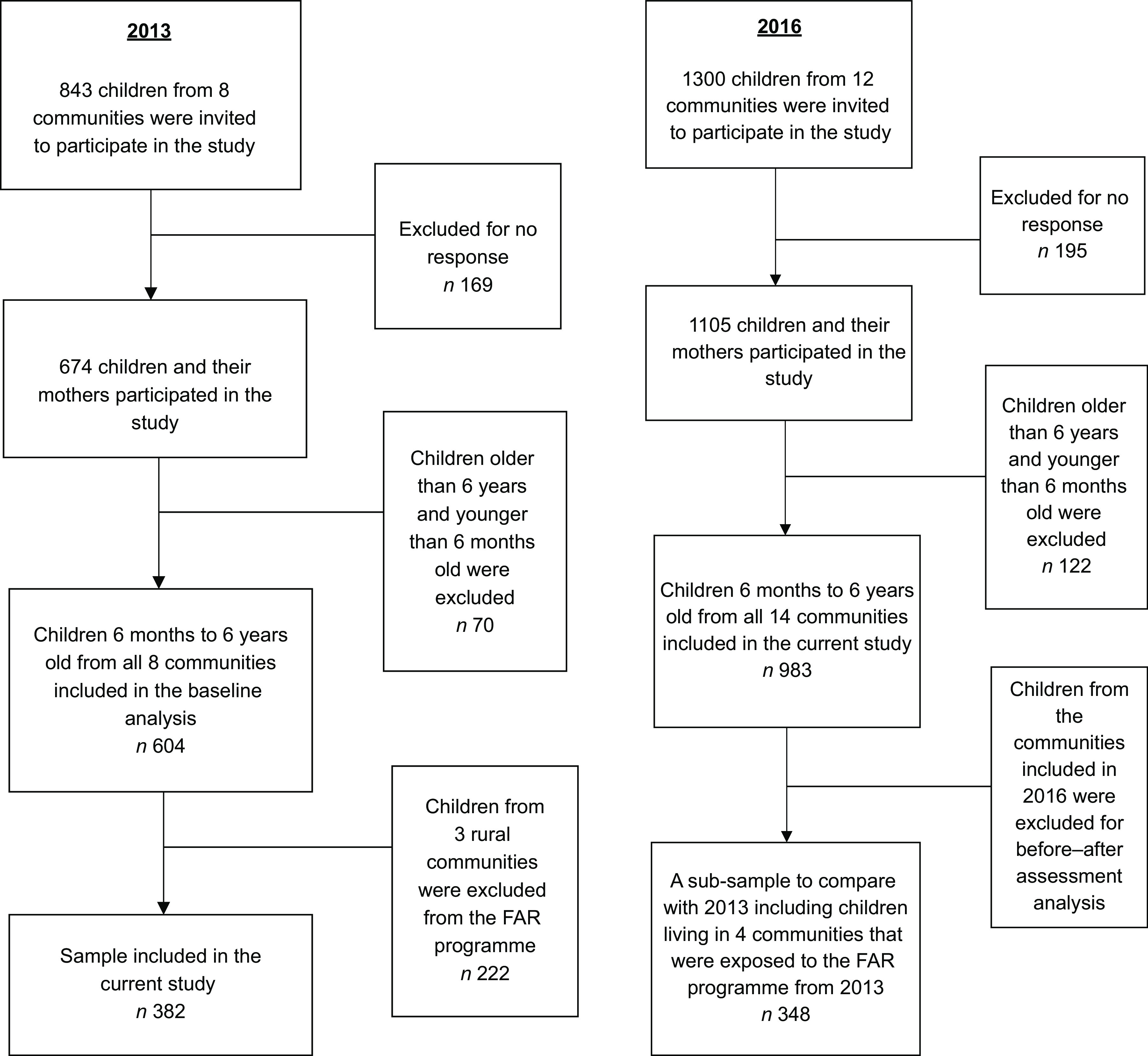
Flowchart of study participant selection in 2013 and 2016 with inclusion and exclusion criteria. We included the data collected from 382 participants of the baseline survey conducted in 2013 in the current study. In 2016, 983 children and their caregivers from fourteen communities participated in the current study (FAR, Fund for Armenian Relief)

The sampling frame in 2013 included all children aged 6 months to 6 years living in Berd city and seven surrounding rural communities. In 2016, we constructed a new sampling frame, which included all eligible children in the same age range from Berd city and thirteen surrounding rural communities. The three communities that were excluded from the programme were not included in the sampling frame in 2016. A representative sample of children from Berd city (*n* 184) and all children from the rural communities (*n* 799) were included in the study, for a total of 983 sampled participants in 2016 (Fig. 2). Study participants were chosen from the list of children registered in Tavush Province’s administrative records. Children from the nine communities unexposed to the intervention served as geographic controls for our analysis.

We selected the children from Berd using a stratified random sampling strategy, with stratification by age group (years). The number of children to be sampled within each age group was calculated to estimate the population prevalence of stunting and anaemia, using the ‘proc power’ procedure in the statistical software package SAS version 9.4 (with *α* = 0·05 and *β* = 0·2), and to detect differences between the groups of children who were exposed to the intervention in the period 2013–2016 in Berd and those in the historical comparison group. Based on the baseline survey, we assumed that the prevalence of stunting would be 10 % in the unexposed group and 5 % in the group exposed to the intervention.

### Intervention

The BCPP included financial and social support for families, improvements to schools and training for human resources in the Berd region. The health-care component of the programme targeted to improve the health status of children and their mothers (Box 1). The health-care intervention had several components. In the nutrition subcomponent, we distributed balanced and high-energy foods including fresh dairy, meat, vegetables and fruits to the kindergartens three times daily. As the children spend about 9 h/d at kindergarten, the food consumed there would account for most of their daily food intake. We trained cooks in the kindergartens to prepare meals based on menus developed in collaboration with nutritional experts at the Ministry of Health of the Republic of Armenia. The pre-school facilities were used to identify the mothers of children and include them in community health education classes focusing on prenatal, infant and child nutrition, breast-feeding, complementary feeding, water and sanitation.

### Measurements

The methods of measuring the main variables were similar in the 2013 baseline study^(^ ^20^ ^)^ and the 2016 data collection. Briefly, children’s Hb levels were measured using the HemoCue^®^ HB 301, a device designed for quick analysis of capillary blood Hb^(^ ^25^ ^,^ ^26^ ^)^. Each child’s weight and height (recumbent length for children younger than 24 months) were measured using electronic scales and measuring boards or stadiometers by physicians at each study site.

We administered the same questionnaire in 2016 as in the 2013 baseline study^(^ ^20^ ^)^ with minor modifications intended to better capture the family’s socio-economic status. The baseline study used an adapted and modified version of the questionnaire used in the 2010 Armenia Demographic and Health Survey^(^ ^27^ ^)^, which was itself based on the Infant and Young Child Feeding Practices questionnaire recommended by the WHO and UNICEF to assess feeding practices, as well as maternal and child characteristics^(^ ^28^ ^,^ ^29^ ^)^. Children’s caregivers participated in a self-administered survey after the collection of blood samples and anthropometric measurements.

### Study variables

We hypothesized that living in the communities where FAR had implemented its multidisciplinary programme would expose study participants to the benefits of the programme. Thus, the primary exposure variable was considered positive if the participant lived in the FAR implementation communities from 2013 to 2016 (five communities, Fig. 1).

Our outcome variables were short- and long-term indicators of malnutrition. The short-term indicators were breast-feeding duration, minimum dietary diversity (an indicator of food diversity) and anaemia. Information regarding breast-feeding practices and duration, as well as minimum dietary diversity, was collected from the caregivers’ survey. Minimum dietary diversity was considered positive if the child had consumed foods from at least four of seven food groups (grains; legumes; dairy; meat; eggs; vitamin A-rich foods; other fruits and vegetables) in the past 24 h. The long-term indicators included stunting and wasting, which were calculated based on children’s anthropometric measurements (Box 2).

Box 2 Operational definitions of the main dietary and growth indicators

1. Malnutrition: deficiencies, excesses or imbalances in a person’s intake of energy and nutrients.
2. Anaemia among children aged 6–59 months: Hb level <110 g/l.
3. Stunting: height-for-age at least 2 sd below the median height-for-age of the WHO child growth standards (low height-for-age).
4. Wasting: weight-for-height at least 2 sd below the median weight-for-height of the WHO child growth standards (low weight-for-height).

Other information collected from the caregiver survey included sociodemographic variables such as child gender, weight and length at birth, parental heights, specific questions related to dietary practices within the 24 h preceding the survey, being exposed to printed public health materials and participating in community trainings.

### Statistical analysis

We performed non-parametric *t* tests to compare means and *χ* ^2^ or non-parametric Fisher’s exact tests, as appropriate, to compare proportions. We selected a sample of study participants in 2016 living in the four communities where the FAR programme was implemented to compare baseline characteristics and main outcome measures with corresponding characteristics available from the 2013 baseline study in the same communities (historical control). We used univariate logistic regression using data collected from the entire study population in 2016 to measure associations between living in a community exposed to the intervention and child growth indicators (stunting and anaemia) compared with geographic controls (data from areas in which the programme was not implemented).

Adjustment for confounding was carried out in two steps. First, we adjusted for all the variables associated with both exposure and outcome at *P* < 0·2 in univariate analyses. In the second step, we added all the clinically and conceptually significant variables to the model. The potential confounding variables were anaemia status (entered for the stunting model), minimum dietary diversity, mother and father’s employment status, presence of a sewage system at the household, diarrhoea reported by caregiver, age by year, weight at birth, length at birth, mother’s height, father’s height, child’s BMI and mother’s education level. We imputed missing data for the following covariates using multiple imputation methods with the fully conditional specification procedure^(^ ^30^ ^)^: length at birth (*n* 72); whether child had ever had diarrhoea (*n* 21); father’s employment status (*n* 15); mother’s education level (*n* 12); mother’s employment (*n* 5); and child’s BMI (*n* 3). All analyses using the imputed database and complete cases were conducted using SAS version 9.4, with statistical significance set at *α* = 0·05. The descriptive analysis was performed using the EasySTAT online statistical application^(^ ^31^ ^)^.

## Results

In total, 843 children were selected to participate in the 2013 baseline survey and 1300 in the 2016 survey. The overall response rates were 80 % in 2013 and 85 % in 2016 (Fig. 2). Logistic regression analyses were conducted with all 983 participants who participated in 2016, including the 348 from communities that received the intervention and 635 children from unexposed communities. The sociodemographic characteristics of study participants in 2016 are presented in Table 1.

**Table 1:** Comparison of characteristics of study participants in communities receiving and not receiving the community-based Fund for Armenian Relief multidisciplinary intervention: children aged 6 months to 6 years from Berd region, Armenia (a chronic conflict setting), 2016

Variable†	Total (*n* 983)		Intervention (*n* 347)		No intervention (*n* 636)		*P* value‡
	Mean or *n*	sd or %	Mean or *n*	sd or %	Mean or *n*	sd or %	
Hb (g/l)	118·38	13·34	122·97	11·30	115·87	13·70	0·001
Weight at birth (g)	3124·32	474·56	3141·61	473·72	3115·02	475·13	0·41
Length at birth (cm)	49·61	2·11	49·68	2·01	49·57	2·16	0·46
Mother’s height (cm)	159·98	6·36	160·35	6·53	159·78	6·25	0·18
Father’s height (cm)	169·94	7·04	170·74	6·57	169·55	7·23	0·02
Child’s current age (months)	39·46	19·10	39·98	18·77	39·17	19·28	0·52
Child’s current BMI (kg/m^2^)	15·88	2·21	16·17	2·19	15·72	2·21	0·01
Gender							
Female	515	52·39	177	51·01	338	53·14	0·52
Male	468	47·61	170	48·99	298	46·86	
Kindergarten attendance	10	1·02	4	1·15	6	0·94	
No	469	47·71	165	47·55	304	47·80	0·96
Yes	504	51·27	178	51·30	326	51·26	
Child’s history of sleeping hungry reported by caregiver	3	0·31	1	0·29	2	0·31	0·76
Always	10	1·02	4	1·15	6	0·94	
Never	877	89·22	313	90·20	564	88·68	
Often	5	0·51	1	0·29	4	0·63	
Sometimes	88	8·95	28	8·07	60	9·43	
Mother’s education	12	1·22	3	0·86	9	1·42	0·0001
Higher education	142	14·45	62	17·87	80	12·58	
Incomplete secondary school	109	11·09	25	7·20	84	13·21	
Secondary school	464	47·20	131	37·75	333	52·36	
Vocational secondary school	256	26·04	126	36·31	130	20·44	
Mother’s employment							
Missing	5	0·51	3	0·86	2	0·31	0·16
Maternity leave	56	5·70	25	7·20	31	4·87	
No	758	77·11	256	73·78	502	78·93	
Yes	164	16·68	63	18·16	101	15·88	
Father’s employment							
Missing	15	1·53	11	3·17	4	0·63	0·0001
No	349	35·50	83	23·92	266	41·82	
Other	16	1·63	5	1·44	11	1·73	
Yes	603	61·34	248	71·47	355	55·82	
Monthly expenditures (AMD)	18	1·83	6	1·73	12	1·89	
<50 000	265	26·96	74	21·33	191	30·03	0·01
51 000–100 000	392	39·88	161	46·40	231	36·32	
101 000–200 000	251	25·53	85	24·50	166	26·10	
201 000–300 000	45	4·58	18	5·19	27	4·25	
>301 000	12	1·22	3	0·86	9	1·42	
Presence of sewage system at residence	15	1·53	9	2·59	6	0·94	
No	390	39·67	123	35·45	267	41·98	0·07
Yes	578	58·80	215	61·96	363	57·08	
Participating in community training	4	0·41	3	0·86	1	0·16	
No	397	40·39	120	34·58	277	43·55	0·008
Yes	582	59·21	224	64·55	358	56·29	
Receiving printed materials	6	0·61	3	0·86	3	0·47	0·83
No	249	25·33	89	25·65	160	25·16	
Yes	728	74·06	255	73·49	473	74·37	
Minimum dietary diversity							
No	154	15·67	73	21·04	81	12·74	<0 001
Yes	829	84·33	274	78·96	555	87·26	
Anaemia status							
Anaemic	237	24·11	37	10·66	200	31·45	<0·001
Non-anaemic	746	75·89	310	89·34	436	68·55	
Stunting status							
Not stunted	894	90·95	307	88·47	587	92·30	0·04
Stunted	89	9·05	40	11·53	49	7·70	
Wasting status							
Wasted	39	3·97	33	3·36	6	3·11	0·69
Not wasted	943	95·93	756	95·70	187	96·89	

AMD, Armenian Drams (currency).

† Continuous variables are presented as mean and standard deviation; categorical variables are presented as number and percentage.

‡ For continuous variables, the *t* test was used. For categorical variables, Pearson’s *χ* ^2^ test was performed if all cell counts were >5. Otherwise, Fisher’s exact test was performed. *P* values are derived from the *t* test for equality of means for continuous variables and the *χ* ^2^ test of equality of proportions for categorical variables between the groups exposed/not exposed to the intervention 2013–2016.

The children living in the communities that were not exposed to the FAR programme from 2013 to 2016 had a lower prevalence of stunting (7·70 *v*. 11·53 %); a higher prevalence of anaemia (31·45 *v*. 10·66 %); higher maternal educational attainment (52·36 *v*. 37·75 % completed secondary school); and a higher prevalence of paternal unemployment (41·82 *v*. 23·92 %).

Table 2 shows a comparison of demographic characteristics and study outcomes between study participants residing in the communities where FAR had implemented its multidisciplinary programme in 2016 (*n* 348) and those living in the same communities during the baseline assessment in 2013 (*n* 382). Demographic characteristics were similar in the 2013 and 2016 samples.

**Table 2:** Comparison of characteristics of study participants in communities before (2013) and after the community-based Fund for Armenian Relief multidisciplinary intervention (2016): children aged 6 months to 6 years from Berd region, Armenia (a chronic conflict setting)

Variable†	Before intervention‡ (*n* 382)		After intervention‡ (*n* 348)		*P* value§
	Mean or *n*	sd or %	Mean or *n*	sd or %	
BMI (kg/m^2^)	16·76	2·41	16·17	2·18	0·0001
Breast-feeding duration (months)	11·47	6·91	12·98	6·52	0·002
Mother’s height (cm)	159·90	5·92	160·36	6·52	0·32
Father’s height (cm)	169·90	6·87	170·80	6·56	0·10
Length at bir†h (cm)	49·73	2·23	49·67	2·01	0·73
Weight at birth (g)	3151·40	481·20	3141·19	473·08	0·77
Gender					
Female	188	49·21	178	51·15	0·60
Male	194	50·79	170	48·85	
Age					
<24 months	112	29·32	86	24·71	0·16
≥24 months	270	70·68	362	75·29	
Residence					
Rural	174	45·55	163	46·84	0·73
Urban	208	54·45	185	53·16	
Mother’s education					
Incomplete secondary	14	3·66	25	7·18	1
Secondary	181	47·38	131	37·64	1
Vocational	147	38·48	126	36·21	0·65
Higher	38	9·95	63	18·10	0·67
Minimum dietary diversity					
No	122	31·94	73	20·98	0·001
Yes	260	68·06	275	79·02	
Child’s history of sleeping hungry reported by the caregiver					
Never	297	77·75	314	90·49	0·0001
Ever	78	20·42	33	9·51	
Caregiver reported diarrhoea					
No	310	81·15	295	84·77	
Yes	67	17·54	48	13·79	
Participating in community training					
No	313	81·94	121	34·77	0·0001
Yes	64	16·75	224	64·37	
Receiving printed materials					
No	273	71·47	8	25·57	0·0001
Yes	102	26·70	256	73·56	
Stunting					
Not stunted	348	89·79	308	88·51	0·57
Stunted	39	10·21	40	11·49	
Anaemia					
Non-anaemic	308	80·84	310	89·08	0·002
Anaemic	73	19·06	38	10·92	
Wasting					
Not wasted	288	97·96	259	95·22	0·07
Wasted	6	2·04	13	4·78	

† Continuous variables are presented as mean and standard deviation; categorical variables are presented as number and percentage.

‡ The communities compared before and after the intervention are Tavush, Aygepar, Nerkin Karmiraghbyur, Berd and Chinchin.

§ *P* values are derived from the the *t* test for equality of means for continuous variables and the *χ* ^2^ test of equality of proportions for categorical variables between the groups before/after the intervention 2013–2016.

### Anaemia and short-term dietary indicators of malnutrition

The prevalence of anaemia was significantly lower in 2016 than in 2013 (19·1 % in 2013 *v*. 10·9 % in 2016, *P* = 0·002). Compared with children from the baseline assessment in 2013, more children included in the 2016 sample had consumed at least four different food groups in the past 24 h (minimum dietary diversity; 79·0 *v*. 68·1 %, *P* < 0·001). The duration of breast-feeding was also significantly higher in 2016 compared with 2013 (13·0 *v*. 11·5 months, *P* < 0·002) and the percentage of mothers reporting their children had gone to sleep hungry was significantly lower (9·5 *v*. 20·4 %, *P* < 0·0001; Table 2).

The anaemic children were more likely to have slightly higher BMI and to have been breast-fed for a slightly shorter period than non-anaemic children. The prevalence of anaemia was significantly higher in rural than in urban areas (26·28 *v*. 14·67 %, *P* < 0·001; see online supplementary material, Supplemental Table S1). The other social and demographic characteristics examined did not differ significantly between anaemic and non-anaemic children. In crude logistic regression analyses, the odds of anaemia were 74 % lower in intervention communities compared with communities not receiving the intervention (OR =0·26; 95 % CI 0·18, 0·38). These effect estimates were not affected after adjusting for potential social and demographic confounders (OR = 0·24; 95 % CI 0·16, 0·36; Table 3, model E).

**Table 3:** Estimated odds of stunting and anaemia for children aged 6 months to 6 years living in communities in 2016 where the community-based Fund for Armenian Relief (FAR) multidisciplinary intervention was implemented, Berd region, Armenia (a chronic conflict setting)

	Stunting (*n* 983)						Anaemia (*n* 983)					
	Crude model		Model B		Model C		Crude model		Model D		Model E	
	OR	95 % CI	OR	95 % CI	OR	95 % CI	OR	95 % CI	OR	95 % CI	OR	95 % CI
Communities where FAR was not present (2013–2016)	Ref.		Ref.		Ref.		Ref.		Ref.		Ref.	
Communities where FAR was present (2013–2016)	1·56	1·005, 2·42	1·92**	1·18, 3·13	1·92*	1·13, 3·26	0·26*	0·18, 0·38	0·24***	0·16, 0·37	0·24***	0·16, 0·36

Ref., reference category.

Model B: crude model for stunting adjusted for anaemia, minimum dietary diversity, mother’s and father’s employment status, having a sewage system at the household, caregiver’s reported child diarrhoea, and age by year.

Model C: model B plus adjusted for weight at birth, length at birth, mother’s height, father’s height, child’s BMI, mother’s education level, and age by year.

Model D: crude model for anaemia adjusted for caregiver’s reported child diarrhoea, mother and father’s employment status, mother’s education level, length at birth, BMI, minimum dietary diversity, and age by year.

Model E: model D plus adjusted for weight at birth, having sewage system at the household and child’s kindergarten attendance reported by the caregiver.

*P < 0·05, **P < 0·01, ***P < 0·001.

### Long-term indicators of malnutrition

The prevalence of stunting and wasting did not differ significantly between the two samples (10·2 % in 2013 *v*. 11·5 % in 2016, *P* = 0·57 for stunting; 2·0 *v*. 4·8 %, *P* = 0·07 for wasting; Table 2). However, there were significant differences in BMI, breast-feeding duration, weight and height at birth, and mean maternal and paternal heights in stunted *v*. not stunted children (see online supplementary material, Supplemental Table S2). In crude logistic regression analyses, the odds of being stunted were 56 % higher in communities where the programme was implemented compared with communities not receiving the intervention (OR = 1·56; 95 % CI 1·005, 2·42; Table 3). In the multivariable model adjusted for social and demographic covariates (model C, Table 3), the odds of being stunted were significantly higher for the children living in communities exposed to the intervention (OR = 1·92; 95 % CI 1·13, 3·26). No significant difference in effect estimates was observed between the imputed and complete-case databases (Supplemental Table S3).

## Discussion

Our study found that communities receiving a multidisciplinary intervention experienced significant changes in short-term indicators of nutrition and child growth including anaemia, breast-feeding practices, minimum dietary diversity and reports of going to sleep hungry. Our findings are in line with the observed effects of other interventions around the globe aimed at fighting malnutrition^(^ ^22^ ^,^ ^32^ ^–^ ^35^ ^)^. For example, the prevalence of anaemia decreased significantly following implementation of a community-level fortification programme in Costa Rica^(^ ^36^ ^)^. Hb concentration was increased by 2·0 g/l in a recent cluster-randomized trial among children who received small food packages as an intervention^(^ ^37^ ^)^. A meta-analysis of the effect of dietary interventions on iron-deficiency anaemia also found a positive impact for such interventions^(^ ^38^ ^)^.

In multivariable logistic regression analyses, we found that the odds of being anaemic were lower in communities exposed to the intervention than in unexposed communities. We found a higher proportion of children with positive minimum dietary diversity (consumption from at least four food groups in the past 24 h) as well as longer breast-feeding duration in communities exposed to the intervention.

The improvement in these short-term indicators of nutrition status could itself have resulted in the reduction of the prevalence of anaemia. The increase in the proportion of children meeting minimum dietary diversity could indicate an improvement in the overall socio-economic status of communities included in the intervention. Similarly, the increase in the proportion of the children who never slept hungry (as reported by the caregiver) could be considered another indicator of improved socio-economic status. Higher socio-economic status has been linked with higher dietary diversity and breast-feeding status in several lower-middle-income countries^(^ ^39^ ^–^ ^44^ ^)^.

The BCPP intervention trained mothers about the significance of breast-feeding and complementary feeding, and also included distribution of balanced, high-energy nutrition in kindergartens. The distributed food included at least four food groups, meeting the requirements for minimum dietary diversity. Since breast milk alone is not sufficient to support the nutritional needs of the growing child after 6 months of age^(^ ^45^ ^)^, timely introduction of complementary feeding with breast milk is crucial for further growth and development of children.

We found a non-significantly higher prevalence of stunting in the subset of the 2016 sample compared with data from 2013 (historical controls). This finding was reconfirmed in parametric logistic regression analysis of the entire study population in 2016, comparing children in the intervention communities with those in communities that were not included in the programme in 2013 (geographic controls). The higher odds of being stunted in intervention communities persisted after the model was adjusted for potential confounders.

Our finding of no significant change in the prevalence of stunting was in agreement with those from previous studies conducted in South Africa^(^ ^46^ ^)^, Brazil^(^ ^47^ ^)^ and Palestine^(^ ^48^ ^)^, as well as a meta-analysis^(^ ^49^ ^)^ finding that food distribution programmes had little or no effect on improving height-for-age over similar time frames.

Similar findings were also reported when the effects of interventions were assessed over a shorter time span. A study in Indonesia evaluating the effect of fortified infant foods found an increase in the prevalence of stunting at 12 months compared with 6 months^(^ ^50^ ^)^. Studies in Bhutan, Nepal, Kenya and Bangladesh found that the prevalence of stunting was decreased in response to micronutrient powder distribution programmes^(^ ^51^ ^,^ ^52^ ^)^. However, the duration of these studies was longer than our study. Moreover, the difference in the type of intervention (i.e. micronutrient powders *v*. a pre-school feeding programme) could be responsible for the discrepancy in results.

We found higher odds of being stunted among children living in the FAR intervention communities compared with unexposed communities in 2016. This finding may partially stem from surveillance bias among physicians residing in communities served by the programme: physicians in the intervention communities may have performed a more rigorous examination of children’s growth indicators in 2016 than in 2013. Surveillance bias may also be due to the extensive training physicians received during implementation of the multidisciplinary programme, which may have led to more accurate readings in 2016 than at baseline. In contrast, the Hb measurement was performed with a standardized machine, which would minimize the risk of subjective or biased measurements in all communities.

Interestingly, the geographic controls that were not exposed to the nutrition programme during 2013–2016 tended to have a lower prevalence of stunting and a lower proportion of children who slept hungry at night (Table 1). The lower prevalence of stunting among this group could imply that the children in these communities were initially generally healthier than those in the intervention communities. However, a lack of baseline data from 2013 on geographic control communities limits our ability to interpret the reasons for this difference.

Another possible explanation for the higher prevalence of stunting observed in the intervention communities is that stunting is an indicator of chronic malnutrition, which is likely more difficult to modify than the other outcomes we measured and may not be sensitive to interventions within a short time span^(^ ^35^ ^,^ ^53^ ^)^. However, it has been shown that multidisciplinary programmes can combat stunting over a longer time period^(^ ^54^ ^)^. This finding was confirmed in studies in Malawi, Ethiopia and Haiti evaluating a community-based programme addressing stunting over a period of 5–8 years^(^ ^55^ ^–^ ^57^ ^)^.

### Strengths

To our knowledge, the present study was the first to evaluate the effects of a multidisciplinary community-based programme addressing child malnutrition in a frozen conflict setting. The principal strength of the study is the use of a census approach to cover all children in rural communities aged 6 months to 6 years. Although the communities were situated in a conflict area, we presume that very little or no migration took place over the study period, as the programme was improving living conditions and reducing the burden of feeding children. These low levels of migration add to the comparability of the 2013 and 2016 study populations. While the two populations sampled were essentially different, our sampling strategy led the two study groups to be comparable and representative of the same source population, as illustrated by their demographic characteristics.

### Limitations

Our study had a number of limitations. First, although the local study coordinators underwent extensive training on performing anthropometric measurements, the possibility of measurement error cannot be ruled out. Moreover, as described earlier, the measures could be subject to surveillance bias by the study coordinators who were included in the programme from 2013. Second, the study instrument was not validated. Although we utilized the questionnaire used by the Armenian Demographic and Health Survey and the National Statistics Center of Armenia (with minor changes), we did not evaluate the psychometric properties of this questionnaire. This resulted in incomplete information regarding important socio-economic variables including type of employment and household composition. However, we think that such inaccuracies would be similar between the stunted and not-stunted children. Third, the samples collected in 2016 and 2013 were essentially different individuals and three communities dropped out of the intervention. While we acknowledge that there is a possibility of selection bias, the nature of the intervention was such that identical communities were selected. All communities included in the study were very similar to each other in terms of sociodemographic characteristics such as their ethnicity, culture, beliefs, socio-economic status and practices, which may have otherwise had the potential to introduce bias. However, a lack of randomized intervention allocation, as well as temporal follow-up of the study participants, limits the causal interpretation of the changes in the prevalence of stunting and its attribution to the intervention. Finally, information collected about children’s feeding practices and health indicators, such as diarrhoea, was reported by caregivers and is subject to recall bias and caregivers’ perception of their children’s health.

## Conclusion

Short-term nutritional and child growth outcomes improved in response to a multidisciplinary intervention programme implemented in a frozen conflict setting. This programme may serve as a model for replication in similar conflict zone settings, providing guidance for effective collaboration with government and local health-care professionals to improve children’s dietary status and growth. However, understanding the long-term impact and effectiveness of such programmes will require future studies of other developmental measures, including cognitive, motor, emotional and behavioural milestones.

## Supplementary material

For supplementary material accompanying this paper visit https://doi.org/10.1017/S1368980019002969. click here to view supplementary material
