# Supplementary material for: Short-term nutrition and growth indicators in 6-month- to 6-year-old children are improved following implementation of a multidisciplinary community-based programme in a chronic conflict setting
Source: Public Health Nutr. 2019 Nov 7;23(1):134–45. doi: 10.1017/S1368980019002969 (PMC6958560; doi:10.1017/S1368980019002969)
Supplement: Supplementary file 1 [file S1368980019002969sup001.docx]

**Supplemental Table 1. Comparison of Sociodemographic variables by Stunting in all the 14 communities included in FAR program**

| **Variable** | **Level** | **Total N=983  (%, mean +/- SD)** | **Not Stunted N=894** | **Stunted N=89** | **p value** |
| --- | --- | --- | --- | --- | --- |
| **BMI (mean +/- SD)** |  | 15.88 (+/- 2.21) | 15.71 (+/- 2.04) | 17.59 (+/- 3.01) | 0.001 |
| **Breastfeeding Duration (mean +/- SD)** |  | 12.62 (+/- 6.57) | 12.74 (+/- 6.64) | 11.32 (+/- 5.72) | 0.0373 |
| **Mohter’s height (mean +/- SD)** |  | 159.98 (+/- 6.36) | 160.19 (+/- 6.35) | 157.86 (+/- 6.04) | 0.001 |
| **Father’s height (mean +/- SD)** |  | 169.94 (+/- 7.04) | 170.29 (+/- 7.02) | 166.39 (+/- 6.32) | 0.001 |
| **Weight at birth (cm) (mean +/- SD)** |  | 3124.32 (+/- 474.56) | 3139.21 (+/- 475.18) | 2974.94 (+/- 443.83) | 0.001 |
| **Length at birth (cm) (mean +/- SD)** |  | 49.61 (+/- 2.11) | 49.67 (+/- 2.11) | 48.98 (+/- 1.99) | 0.003 |
| **Hemoglobin (mean +/- SD)** |  | 118.38 (+/- 13.34) | 118.77 (+/- 12.96) | 114.4 (+/- 16.18) | 0.0153 |
| **Gender (n,%)** | Female | 515 (52.39%) | 467 (90.68%) | 48 (9.32%) | 0.76 |
|  | Male | 468 (47.61%) | 427 (91.24%) | 41 (8.76%) |  |
| **Residence (n,%)** |  |  |  |  | 0.34 |
|  | Urban | 184 (18.70) | 164(89.13) | 20 (10.87) |  |
|  | Rural | 799 (81.30) | 730 (91.36) | 69 (8.63) |  |
| **Mother’s education (n,%)** |  |  |  |  | 0.65 |
|  | Missing | 12 (1.22%) | 9 (75%) | 3 (25%) |  |
|  | Higher education | 142 (14.45%) | 131 (92.25%) | 11 (7.75%) |  |
|  | Incomplete secondary school | 109 (11.09%) | 96 (88.07%) | 13 (11.93%) |  |
|  | Secondary school | 464 (47.2%) | 423 (91.162%) | 41 (8.84%) |  |
|  | Vocational secondary school | 256 (26.04%) | 235 (91.80%) | 21 (8.20%) |  |
| **Child’s history of sleeping hungry reported by caregiver (n,%)** |  |  |  |  | 0.54 |
|  | Missing | 3 (0.31%) | 3 (100%) | 0 (0%) |  |
|  | always | 10 (1.02%) | 8 (80%) | 2 (20%) |  |
|  | Never | 877 (89.22%) | 799 (91.11%) | 78 (8.89%) |  |
|  | often | 5 (0.51%) | 5 (100%) | 0 (0%) |  |
|  | Sometimes | 88 (8.95%) | 79 (89.97%) | 9 (10.23%) |  |
| **History of Diarrhea reported by the caregiver (n,%)** |  |  |  |  | 0.11 |
|  | Missing | 21 (2.14%) | 20 (95.24%) | 1 (4.76%) |  |
|  | no | 807 (82.1%) | 728 (90.21%) | 79 (9.79%) |  |
|  | yes | 155 (15.77%) | 146 (94.19%) | 9 (5.81%) |  |
| **Mother’s employment (n,%)** |  |  |  |  | 0.04 |
|  | Missing | 5 (0.51%) | 5 (100%) | 0 (0%) |  |
|  | maternity leave | 56 (5.7%) | 52 (92.86%) | 4 (7.14%) |  |
|  | no | 758 (77.11%) | 680 (89.71%) | 78 (10.29%) |  |
|  | yes | 164 (16.68%) | 157 (95.73%) | 7 (4.27%) |  |
| **Father’s employment (n,%)** |  |  |  |  | 0.10 |
|  | Missing | 15 (1.53%) | 15 (100%) | 0 (0%) |  |
|  | no | 349 (35.5%) | 309 (88.54%) | 40 (11.46%) |  |
|  | other | 16 (1.63%) | 16 (100%) | 0 (0%) |  |
|  | yes | 603 (61.34%) | 554 (91.87%) | 49 (8.13%) |  |
| **Monthly expenditures (n,%)** |  |  |  |  | 0.43 |
|  | Missing | 18 (1.83%) | 16 (88.89%) | 2 (11.11%) |  |
|  | < 50,000 | 265 (26.96%) | 237 (89.43%) | 28 (31.46%) |  |
|  | 51,000 - 100,000 | 392 (39.88%) | 363 (92.60%) | 29 (7.40%) |  |
|  | 101,000 - 200,000 | 251 (25.53%) | 225 (89.64%) | 26 (10.36%) |  |
|  | 201,000 - 300,000 | 45 (4.58%) | 41 (91.11%) | 4 (8.89%) |  |
|  | > 301,000 | 12 (1.22%) | 12 (100%) | 0 (0%) |  |
| **Presence of sewage system at home (n,%)** |  |  |  |  | 0.18 |
|  | Missing | 15 (1.53%) | 11 (73.33) | 4 (26.67%) |  |
|  | No | 390 (39.67%) | 350 (89.74%) | 40 (10.26%) |  |
|  | Yes | 578 (58.8%) | 533 (92.21%) | 45 (7.79%) |  |
| **Participating in community training (n,%)** |  |  |  |  | 0.84 |
|  | Missing | 4 (0.41%) | 4 (100%) | 0 (0%) |  |
|  | No | 397 (40.39%) | 360 (90.68%) | 37 (9.32%) |  |
|  | Yes | 582 (59.21%) | 530 (91.07%) | 52 (8.93%) |  |
| **Receiving printed materials (n,%)** |  |  |  |  | 0.91 |
|  | Missing | 6 (0.61%) | 5 (83.33%) | 1 (16.67%) |  |
|  | No | 249 (25.33%) | 227 (91.16%) | 22 (8.84%) |  |
|  | Yes | 728 (74.06%) | 662 (90.93%) | 66 (9.07%) |  |
| **Minimum Dietary Diversity** |  |  |  |  | 0.215 |
|  | no | 154 (15.67%) | 136 (88.31%) | 18 (11.56%) |  |
|  | yes | 829 (84.33%) | 758 (91.44%) | 71 (8.56%) |  |
| **Anemia** (n,%) |  |  |  |  | 0.0891 |
|  | Anemic | 237 (24.11%) | 209 (88.19%) | 28 (8.18%) |  |
|  | Non Anemic | 746 (75.89%) | 685 (91.82%) | 61 (11.81%) |  |

**Supplemental Table 2. Comparison of Sociodemographic variables by Anemia in all the 14 communities included in FAR program**

| **Variable** | **Level** | **Total N=983  (%, mean +/- SD)** | **Anemic N=237** | **Non Anemic N=746** | **p value** |
| --- | --- | --- | --- | --- | --- |
|  |  |  |  |  |  |
| **BMI (mean +/- SD)** |  | 15.88 (+/- 2.21) | 16.23 (+/- 2.49) | 15.77 (+/- 2.1) | 0.004 |
| **Breastfeeding Duration (mean +/- SD)** |  | 12.62 (+/- 6.57) | 11.91 (+/- 5.95) | 12.85 (+/- 6.75) | 0.0469 |
| **Mother’s height (mean +/- SD)** |  | 159.98 (+/- 6.36) | 159.65 (+/- 6.31) | 160.09 (+/- 6.37) | 0.356 |
| **Father’s height (mean +/- SD)** |  | 169.94 (+/- 7.04) | 170.18 (+/- 7.15) | 169.86 (+/- 7.01) | 0.569 |
| **Weight at birth (cm) (mean +/- SD)** |  | 3124.32 (+/- 474.56) | 3081.88 (+/- 446.3) | 3138 (+/- 482.82) | 0.102 |
| **Length at birth (cm) (mean +/- SD)** |  | 49.61 (+/- 2.11) | 49.52 (+/- 1.9) | 49.64 (+/- 2.17) | 0.50 |
| **Gender (n,%)** |  |  |  |  | 0.746 |
|  | Female | 515 (52.39%) | 122 (23.69%) | 393 (76.31%) |  |
|  | Male | 468 (47.61%) | 115 (24.57%) | 353 (75.43%) |  |
| **Residence (n,%)** |  |  |  |  | 0.00 |
|  | Urban | 184 (18.70) | 27 (14.67%) | 157 (85.33%) |  |
|  | Rural | 799 (81.30) | 210 (26.28%) | 589 (73.72%) |  |
| **Child’s history of sleeping hungry reported by caregiver (n,%)** |  |  |  |  |  |
|  | Missing | 3 (0.31%) | 0 (0%) | 3 (100%) | 0.86 |
|  | Always | 10 (1.02%) | 3 (30%) | 7 (70%) |  |
|  | Never | 877 (89.22%) | 209 (23.83%) | 668 (76.17%) |  |
|  | Often | 5 (0.51%) | 1 (20%) | 4 (80%) |  |
|  | Sometimes | 88 (8.95%) | 24 (27.27%) | 64 (72.73%) |  |
| **History of Diarrhea reported by the caregiver (n,%)** |  |  |  |  | 0.18 |
|  | Missing | 21 (2.14%) | 4 (19.05%) | 17 (80.95%) |  |
|  | no | 807 (82.1%) | 189 (23.42%) | 618 (76.58%) |  |
|  | yes | 155 (15.77%) | 44 (28.39%) | 111 (71.61%) |  |
|  |  |  |  |  |  |
| **Mother’s education (n,%)** | Missing | 12 (1.22%) | 3 (25%) | 9 (75%) | 0.11 |
|  | higher education | 142 (14.45%) | 25 (17.61%) | 117 (82.39%) |  |
|  | Incomplete secondary school | 109 (11.09%) | 29 (26.61%) | 80 (73.39%) |  |
|  | secondary school | 464 (47.2%) | 124 (26.72%) | 340 (73.28%) |  |
|  | vocational secondary school | 256 (26.04%) | 56 (21.88%) | 200 (78.13%) |  |
|  |  |  |  |  |  |
| **Mother’s employment (n,%)** | Missing | 5 (0.51%) | 1 (20%) | 4 (80%) | 0.02 |
|  | maternity leave | 56 (5.7%) | 16 (28.57%) | 40 (71.43%) |  |
|  | no | 758 (77.11%) | 198 (26.12%) | 560 (73.88%) |  |
|  | yes | 164 (16.68%) | 22 (13.41%) | 142 (86.59%) |  |
|  |  |  |  |  |  |
| **Father’s employment (n,%)** | Missing | 15 (1.53%) | 2 (13.33%) | 13 (86.67%) | 0.51 |
|  | no | 349 (35.5%) | 92 (26.36%) | 257 (73.64%) |  |
|  | other | 16 (1.63%) | 4 (25%) | 12 (75%) |  |
|  | yes | 603 (61.34%) | 139 (23.05%) | 464 (76.95%) |  |
|  |  |  |  |  |  |
| **Monthly expenditures (n,%)** | Missing | 18 (1.83%) | 2 (11.11%) | 16 (88.89%) | 0.86 |
|  | < 50,000 | 265 (26.96%) | 65 (24.53%) | 200 (75.47%) |  |
|  | 51,000 - 100,000 | 392 (39.88%) | 99 (25.26%) | 293 (74.74%) |  |
|  | 101,000 - 200,000 | 251 (25.53%) | 56 (22.31%) | 195 (77.69%) |  |
|  | 201,000 - 300,000 | 45 (4.58%) | 11 (24.44%) | 34 (75.56%) |  |
|  | > 301,000 | 12 (1.22%) | 4 (33.33%) | 8 (66.67%) |  |
| **Presence of sewage system at home (n,%)** |  |  |  |  |  |
|  | Missing | 15 (1.53%) | 3 (20%) | 12 (80%) | 0.84 |
|  | No | 390 (39.67%) | 93 (23.85%) | 297 (76.15%) |  |
|  | Yes | 578 (58.8%) | 141 (24.39%) | 437 (75.61%) |  |
| **Participating in community training (n,%)** |  |  |  |  |  |
|  | Missing | 4 (0.41%) | 1 (25%) | 3 (75%) | 0.57 |
|  | No | 397 (40.39%) | 92 (23.17%) | 305 (76.83%) |  |
|  | Yes | 582 (59.21%) | 144 (24.74%) | 438 (75.26%) |  |
| **Receiving printed materials (n,%)** |  |  |  |  |  |
|  | Missing | 6 (0.61%) | 0 (0%) | 6 (100%) | 0.15 |
|  | No | 249 (25.33%) | 52 (20.88%) | 197 (79.12%) |  |
|  | Yes | 728 (74.06%) | 185 (25.41%) | 543 (74.59%) |  |
| **Minimum Dietary Diversity** |  |  |  |  | 0.228 |
|  | no | 154 (15.67%) | 43 (27.92%) | 111 (72.08%) |  |
|  | yes | 829 (84.33%) | 194 (23.40%) | 635 (76.60%) |  |
|  |  |  |  |  |  |
| **Stunting (n,%)** |  |  |  |  | 0.0891 |
|  | Not Stunted | 894 (90.95%) | 209 (23.38%) | 685 (76.62%) |  |
|  | Stunted | 89 (9.05%) | 28 (31.46%) | 61 (68.54%) |  |

**Supplementary Table 3.** Estimated Odds ratios of stunting and anemia for children 6months-6years old living in communities where multicomponent nutrition program was implemented for observations with no missing data on covariates.

|  | **Stunting** | | | **Anemia** | | |
| --- | --- | --- | --- | --- | --- | --- |
|  | **Crude Model(n=983)** | **Model B**  **(n=771)** | **Model C**  **(n=756)** | **Crude Model**  **(n=983)** | **Model D**  **(n=866)** | **Model E**  **(n=841)** |
|  | **OR (95%CI)** | **OR (95%CI)** | **OR(95%CI)** | **OR (95%CI)** | **OR (95%CI)** | **OR (95%CI)** |
| Living in communities were FAR was present during 2014-2016 | 1.56 (1.005, 2.42) | 2.00^**^ (1.21, 3.29) | 2.41* (1.29, 4.42) | 0.26^*^ (0.18, 0.38) | 0.23^***^ (0.15, 0.36) | 0.25^***^ (0.16, 0.38) |
| Model goodness of fit |  |  |  |  |  |  |
| -2logL | 593.372 | 520.751 | 359.639 | 1027.530 | 839.904 | 821.332 |
| AIC | 597.372 | 546.751 | 401.639 | 1031.530 | 871.904 | 859.332 |
| Hosmer Lemeshow test | N/A | 0.60 | 0.29 | N/A | 0.96 | 0.86 |

Abbrevations: Ref, Reference Category; OR, Odds Ratio; CI Confidence Interval;

^*^P<0.05 ^**^P<0.01 ^***^P<0.001

Model B: Crude Model for stunting adjusted for Anemia, Minimum dietary diversity, Mother and Father employment status, Having a sewage system at the household, Diarrhea reported by caregiver, age by year; Model C: Model B + weight at birth, length at birth, mother’s height, father’s height, child’s BMI, mother’s education level and age by year

Model D: Is the crude model for anemia adjusted for Caregiver’s reported child diarrhea, Mother and Father’s employment status, Mother’s education level, Length at birth, BMI, Minimum dietary diversity, and age by year.

Model E: Model D adjusted for Weight at birth, having sewage system at the household, Child’s kindergarten attendance reported by the caregiver
